# Influence of smoking and socioeconomic status on labor analgesia: a nationwide register-based study in Finland

**DOI:** 10.1007/s00404-024-07647-3

**Published:** 2024-07-19

**Authors:** M. Haapanen, I. Kuitunen, M. Vaajala

**Affiliations:** 1 Department of Gynecology and Obstetrics, Mikkeli Central Hospital, Porrassalmenkatu 35-37, 50100 Mikkeli, Finland; 2 Kuopio Pediatric Research Unit (KUPRU), Institute of Clinical Medicine, University of Eastern Finland, Kuopio, Finland; 3 Department of Pediatrics and Neonatology, Kuopio University Hospital, Kuopio, Finland; 4 Faculty of Medicine and Life Sciences, University of Tampere, Tampere, Finland

**Keywords:** Labor analgesia, Register study, Smoking, Socioeconomic status

## Abstract

**Purpose:**

Labor pain can be treated by medical and non-medical analgesia. Smoking during pregnancy has been shown to increase the incidence of several complications and may influence analgesic effectiveness. Previous studies have linked socioeconomic status to the use of epidurals for labor analgesia. We aimed to determine whether smoking and socioeconomic status influence the use of labor analgesia in Finland.

**Methods:**

From January 1, 2004 to December 31, 2018, we collected data from the national Finnish Medical Birth Register on smoking status, labor analgesia, and socioeconomic status during pregnancy. These categorized variables were presented as absolute numbers and percentages. We included data on singleton pregnancies and excluded any data on pregnancies that missed smoking or socioeconomic status.

**Results:**

71,603 women smoked during the first trimester, 42,079 women continued to smoke after the first trimester, and 641,449 were non-smokers. The four most used labor analgesia were nitrous oxide, epidural, other medical analgesia, and non-medical analgesia. The most frequently used analgesia was nitrous oxide, which was used by 60.8% of the group of smokers after the first trimester, 58.8% of smokers during the first trimester, and 54.5% of non-smokers. There were no substantial differences between socioeconomic status classes and labor analgesia used.

**Conclusion:**

Women who continued smoking after the first trimester used labor analgesia more often than non-smokers. There were no clear differences between socioeconomic status classes and labor analgesia used. These findings highlight the need to reduce maternal smoking during pregnancy, and universal social healthcare systems should promote equality in labor analgesia.

## What does this study add to the clinical work

**Table:** 

Women who smoke used labor analgesia more often than non-smokers and socioeconomic status classes did not affect labor analgesia used. Nitrous oxide and epidural were two most used analgesia methods.	

## Introduction

Labor pain depends on the individual, but it is described as one of the most severe types of physical pain women may experience [1]. There are several ways to relieve labor pain, and these methods can be divided into medical and non-medical analgesia. In high-income countries, the use of medical analgesia during labor is common. Depending on the country, it varies from 49.8% to 93.1% in nulliparous women and from 25.2% to 85.7% in multiparous women [2]. Non-medical methods are also common and are used by approximately 50%–70% of women during labor [3, 4].

Smoking during pregnancy is associated with several complications, such as an increased risk of spontaneous abortion, placental abnormality, birth complications, preterm birth, and low birth weight [5]. It is also a known risk factor for congenital malformations and stillbirth [6] and long-term complications of the offspring such as neurological morbidity [7]. Räisänen et al. showed that smokers during pregnancy had epidural analgesia more often than non-smoking women in Finland during the years 2000–2010 [8]. Moreover, Wang et al. showed that smokers generally experience more severe acute pain than non-smokers and use more opioids after surgery [9].

Several studies have also investigated the relationship between socioeconomic status (SES) and labor analgesia used, particularly epidural analgesia. The results vary among countries with publicly funded healthcare systems, such as Canada, Sweden, and Finland. In Canada, women of higher-SES class were most likely to get an epidural [10] but, conversely, in Sweden, lower-SES-class women were more likely to get epidural as labor analgesia [11]. In the USA, the use of neuraxial analgesia varies between states and based on the study there might be patient and hospital factors explaining the differences [12]. Bamber et al. studied the relationship between ethnicity and used labor analgesia in England and found ethnic disparities in used labor analgesia [13]. In Finland, Räisänen et al. did not find substantial differences between SES classes and epidural analgesia used; however, they did not analyze any other analgesia method than epidural [8]. Recent studies have found that fear of childbirth, which has been found to be more common among certain SES classes, increased the use of epidural and spinal analgesia [14, 15].

This study aimed to evaluate the influence of smoking on the labor analgesia used. We hypothesize that smoking during pregnancy increases the use of labor analgesia. We also aimed to determine whether there were any differences between the SES classes and the labor analgesia used.

## Methods

We conducted a nationwide retrospective register-based study using data from the National Medical Birth Register (MBR). The MBR is maintained by the Finnish Institute for Health and Welfare and has high coverage and quality (the current coverage is nearly 100%) [16, 17]. We included every singleton pregnancy between January 1, 2004 and December 31, 2018. The data was requested in 2020 and due to the register, the possible study period was until the end of 2018. The data was ready for analysis in 2022.

For labor analgesia analysis, we excluded non-singleton pregnancies (*n* = 12,132), elective cesarean section (*n* = 52,876), and out-of-hospital deliveries (*n* = 2301). Pregnancies with multiple fetuses were excluded from the analysis, as they differ from singleton pregnancies in antenatal and labor care.

The MBR contains information on pregnancies, delivery statistics, and perinatal outcomes of all births with a birth weight of ≥ 500 g or a gestational age of ≥ 22 + 0 weeks, including maternal smoking habits. Data on self-reported maternal smoking status during pregnancy is collected in women and child welfare clinics and can be either non-smokers, smoking during the first trimester, smoking after the first trimester, or unknown. Women of unknown smoking status (2.5%) were excluded from the analysis. According to a study by Gissler et al., the reliability of the smoking status data is good (i.e., over 92%) [18].

**Fig. 1:**
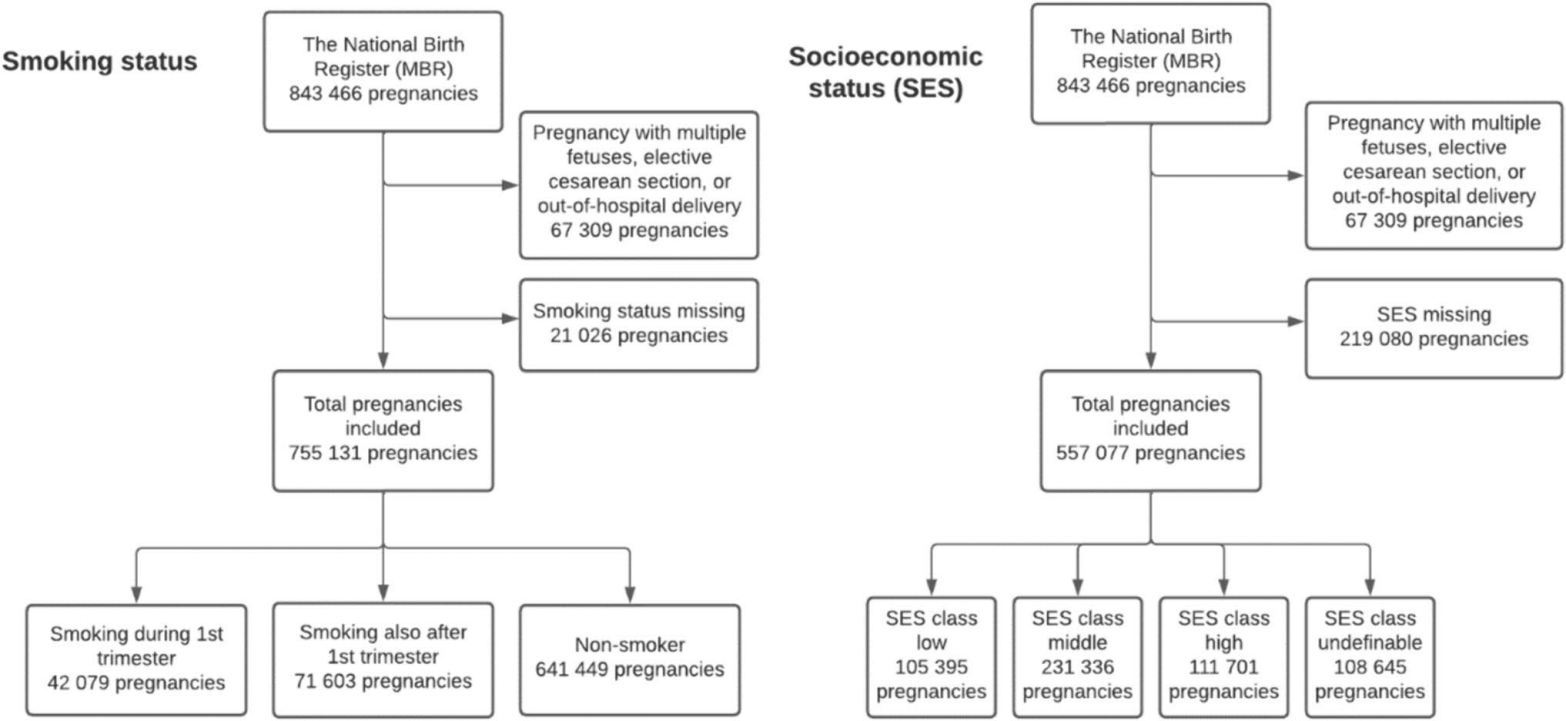
Flowchart of the study groups. Smoking status and socioeconomic status (SES) of the mother during pregnancy were used to form the study groups

Another main variable analyzed in this study was the SES of the mother during pregnancy. We categorized women into four SES classes—low, middle, high, and undefinable—using the SES found in the MBR. The categorization of SES and absolute number with percentages of women in each class is shown in Table 1. Women with missing (26.0%) SES were excluded from the analysis. The flowchart of the study populations with each variable is shown in Fig. 1.

**Table 1:** Categorization of socioeconomic status and total number of patients in each class found in the medical birth register

Class	Specific socioeconomic status
Low	Total number 105,395 (18.9%)
	Agricultural sole proprietors or workers
	Industrial workers
	Other production workers
	Distribution and service representatives
	Indefinite workers
	Other self-employed persons or sole proprietors
	Unemployed (no profession)
	Unemployed (profession coded separately)
	Long-term unemployed
	Retired persons
Middle	Total number 231,336 (41.5%)
	Junior employees in work management position
	Junior employees in independent office work
	Junior employees in non-independent office work
	Other indefinite junior employees
High	Total number 111,701 (20.1%)
	Senior employees in leadership position
	Senior employees in design and research assignments
	Senior employees working in teaching positions
	Other indefinite senior employees
Undefinable or unknown	Total number 108,645 (19.5%)
	Homemaker (taking care of children full-time)
	Students
	Entrepreneurs
	Status coded as unknown

Our main findings related to the use of labor analgesia. The analgesia methods are stratified in the register under the following categories: epidural, spinal, combined epidural and spinal, pudendal, paracervical, nitrous oxide, other medical (including pain medication tablets for example paracetamol, or intravenous opioids), other non-medical (including bathing, aqua vesicles, and TENS), and no analgesia. These are analyzed as categorized (yes or no) variables, as the register does not contain more precise information, for example, on the dosage used.

### Ethics

The National Medical Birth Register (MBR) has a unique pseudonymized identification number for each patient. The pseudonymization was done by the Finnish data authority, Findata. The authors did not have access to the pseudonymization key, as it is maintained by Findata. In accordance with Finnish regulations, no informed written consent was required because of the study’s retrospective register-based study design and because the patients were not contacted. Permission for this data was granted by Findata after evaluation of the study protocol (permission number: THL/1756/14.02.00/2020).

### Statistics

The categorical variables were presented as absolute numbers and percentages- We have used proportional differences (difference of two proportions) with 95% confidence intervals (95% CIs) in the comparisons. In the smoking analysis, we compared the group of smokers to non-smokers and in the SES we compared the other groups to high SES. The results of this study were reported according to the STROBE guidelines [19]. Statistical analysis was performed using R software (version 4.0.3).

## Results

**Fig. 2:**
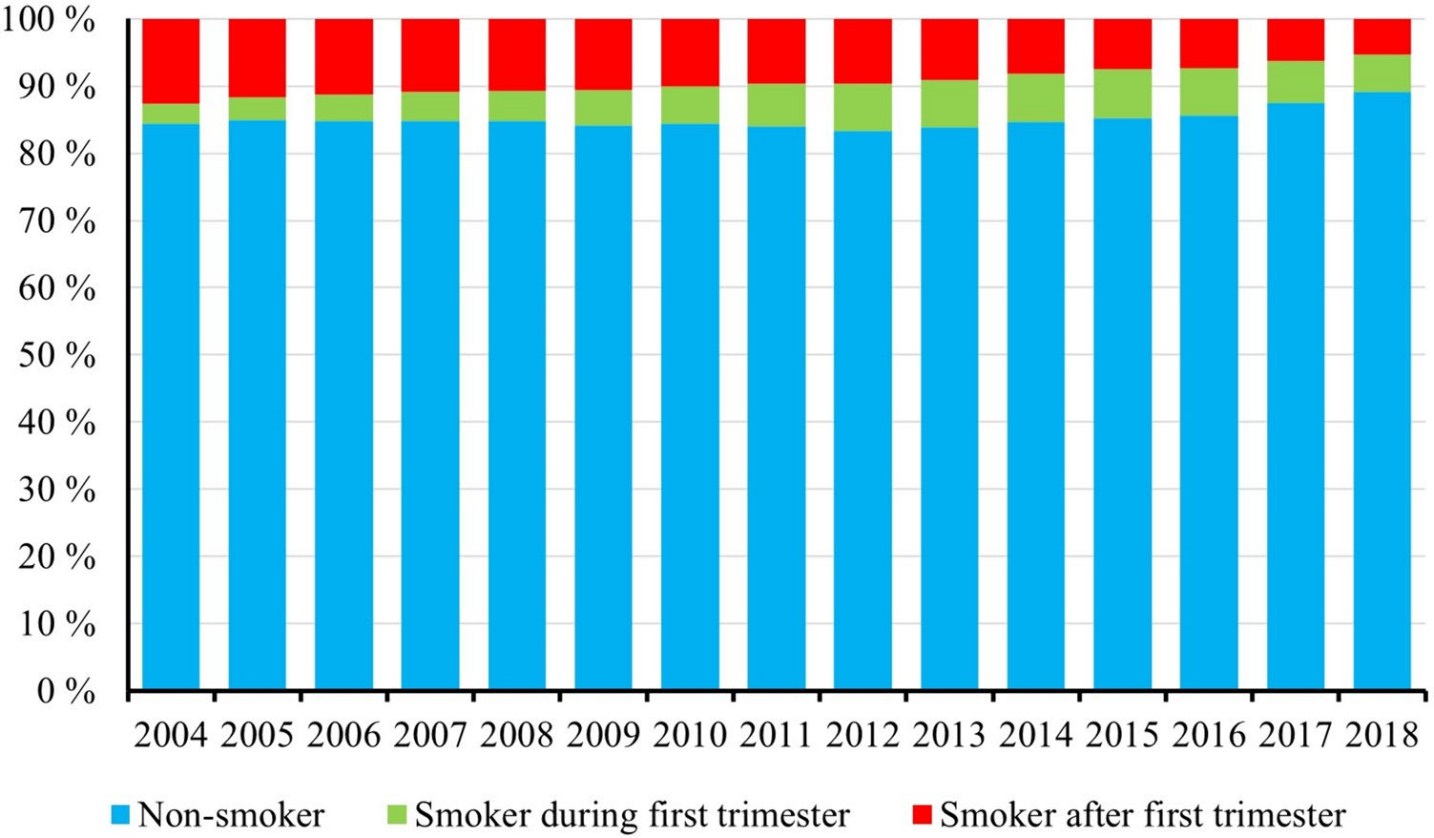
Proportions of women each year who smoked during pregnancy (including after the first trimester), smoked during the first trimester, and did not smoke

According to the MBR, there were 755,131 singleton pregnancies during the study period. Of these, 71,603 women smoked during the first trimester, 42,079 women continued smoking after the first trimester, and 641,449 women did not smoke during pregnancy (Table 2). In addition, 105,395 (18.9%) women of low-SES class, 231,336 (41.5%) women of middle-SES class, 111,701 (20.1%) of high-SES class, and 108,645 (19.5%) of undefinable SES class were identified (Table 1). The middle-SES class was the largest group comprising 41.5% of the patients. During the study period, the annual number of non-smokers varied between 83.3% and 89.0% (Fig. 2). The annual number of patients who continued smoking after the first trimester slowly decreased during the study period, from 12.6% in 2004 to 5.3% in 2018 (Fig. 2).

**Table 2:** Use of labor analgesia among smokers during the first trimester, smokers who continued smoking after the first trimester, and non-smoking women in Finland from 2004 to 2018

Smoking status	Smoker after first trimester		Smoker during first trimester		Non-smoker
Total number of patients (%)	42,079 (5.6)		71,603 (9.5)		641,449 (84.9)
	*n* (%)	Proportional difference (95% CI)^a^	*n* (%)	Proportional difference (95% CI)^a^	*n* (%)
Labor analgesia					
Epidural	24,262 (57.7)	13.5% (13.0–14.0)	35,470 (49.5)	5.4% (5.0–5.8)	283,186 (44.1)
Spinal	5976 (14.2)	1.5% (1.1–1.8)	10,858 (15.2)	0.5% (0.2–0.8)	100,574 (15.7)
Combined spinal and epidural	774 (1.8)	0.1% (0.0–0.3)	1007 (1.4)	0.3% (0.2–0.4)	11,020 (1.7)
Paracervical block	6767 (16.1)	0.3% (0.0–0.7)	11,915 (16.6)	0.2% (0.0–0.5)	105,252 (16.4)
Pudendal block	3564 (8.5)	0.5% (0.2–0.8%	5221 (7.3)	0.7% (0.5–0.9)	51,048 (8.0)
Nitrous oxide	25,594 (60.8)	6.4% (5.9–6.9)	38,489 (53.8)	0.7% (0.3–1.1)	349,319 (54.5)
Other medical analgesia	6686 (15.9)	3.7% (3.3–4.0)	11,626 (16.2)	4.0% (3.7–4.3)	78,356 (12.2)
Non-medical analgesia	15,844 (37.7)	8.5% (8.0–9.0)	17,927 (25.0)	4.1% (3.8–4.5)	187,169 (29.2)
No analgesia	134 (0.3)	0.05% (0.0–0.1)	190 (0.3)	0.1% (0.1–0.1)	2375 (0.4)

Values are numbers (percentages)

^a^The percentage values of smoking groups are compared to the corresponding values of non-smokers

Among all patient groups, the four most-used labor analgesia methods were nitrous oxide, epidural, other medical analgesia, and non-medical analgesia (Table 2). These were also the analgesia types where we saw a difference between smokers and non-smokers. Nitrous oxide was the most used analgesic among all groups. It was used by 60.8% of the group who continued smoking after the first trimester, 53.8% of the group who smoked during the first trimester, and 54.5% of the non-smoking group (Table 2). The proportional difference in the use of nitrous oxide compared to non-smoking group was 6.4% (95% CI 5.9%–6.9%) in the group who smoked after the first trimester and 0.7% (95% CI 0.3%–1.1%) in the group who smoked during the first trimester (Table 2). Epidural analgesia was more used in the smoking groups than in the non-smoking group. It was used by 57.7% of the group who continued smoking after the first trimester, 49.5% of the group who smoked during the first trimester, and 44.1% of the non-smoking group. Other medical analgesia was used by 15.9% and 16.2%, respectively, of the smoking groups and by 12.2% of the non-smoking group. Non-medical analgesia was used by 37.7% and 25.0%, respectively, of the smoking groups and by 29.2% of the non-smoking group. The least used analgesia among all groups was combined spinal and epidural analgesia, and there was a very small percentage of women in all groups that delivered without analgesia (0.3–0.4%) (Table 2).

Overall, the use of labor analgesia was fairly similar between the SES classes (Table 3). Nitrous oxide, epidural, and non-medical analgesia were the three most used labor analgesias, regardless of SES class (Table 3). Among women of high-SES class, the use of spinal analgesia, paracervical block analgesia, other medical analgesia, and non-medical analgesia was slightly lower compared to the other groups (Table 3). For example, spinal analgesia was used by 13.5% of high-SES-class women, 15.6% of low-SES-class women, 16.5% of middle-SES-class women, and 15.1% of undefined-SES-class women (Table 3). The proportional difference in the use of spinal analgesia compared to high-SES class was 2.2% (95% CI 1.9%–2.5%) in the low-SES class, 3.0% (95% CI 2.8%–3.3%) in the middle-SES class and 1.7% (95% CI 1.4%–2.0%) in undefined-SES class (Table 3).

**Table 3:** Use of labor analgesia among women of low, middle, high, and undefinable SES in Finland from 2004 to 2018

Socioeconomic class	Low 105,395		Middle 231,336		High 111,701	Undefined 108,645	
Total number of patients	*n* (%)	Proportional difference (95% CI)^a^	*n* (%)	Proportional difference (95% CI)^a^	*n* (%)	*n* (%)	Proportional difference (95% CI)^a^
Labor analgesia							
Epidural	45,974 (43.6)	0% (0.0–0.4%)	100,012 (43.2)	0.4% (0.0–0.8%)	48,733 (43.6)	47,719 (43.9)	0.3% (0.0–0.7%)
Spinal	16,454 (15.6)	2.2% (1.9–2.5%)	38,077 (16.5)	3.0% (2.8–3.3%)	15,029 (13.5)	16,433 (15.1)	1.7% (1.4–2.0%)
Combined spinal–epidural	853 (0.8)	0.3% (0.2–0.4%)	2044 (0.9)	0.2% (0.2–0.3%)	1259 (1.1)	946 (0.8)	0.3% (0.2–0.3%)
Paracervical block	19,872 (18.9)	3.0% (2.7–3.4%)	42,622 (18.4)	2.6% (2.3–2.9%)	17,670 (15.8)	19,715 (18.1)	2.3% (2.0–2.6%)
Pudendal block	7127 (6.8)	0.1% (0.0–0.3%)	17,140 (7.4)	0.5% (0.4–0.7%)	7672 (6.9)	7056 (6.5)	0.4% (0.2–0.6%)
Nitrous oxide	56,599 (53.7)	0.7% (0.3–1.1%)	125,969 (54.5)	0% (0.0–0.3%)	60,809 (54.4)	55,912 (51.5)	3.0% (2.6–3.4%)
Other medical analgesia	15,433 (14.6)	3.7% (3.4–4.0%)	32,242 (13.9)	3.0% (2.7–3.2%)	12,243 (11.0)	15,138 (13.9)	3.0% (2.7–3.3%)
Non-medical analgesia	30,975 (29.4)	1.0 (0.6–1.4%)	70,291 (30.4)	2.0% (1.7–2.3%)	31,731 (28.4)	32,542 (30.0)	1.6% (1.2–1.9%)
No analgesia	315 (0.3)	0.1% (0.0–0.1%)	727 (0.3)	0.1% (0.0–0.1%)	242 (0.2)	329 (0.3)	0.1% (0.0–0.1%)

Values are numbers (proportions)

^a^The percentage values of different socioeconomic classes are compared to corresponding values of high socioeconomic class

**Fig. 3:**
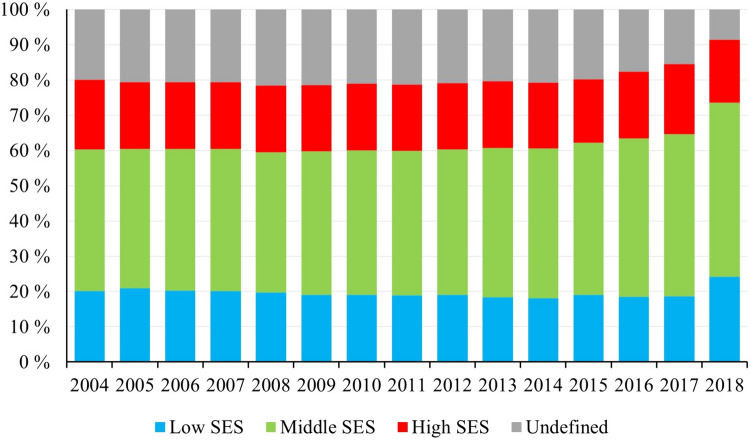
Annual changes in the proportions of pregnant women stratified by socioeconomic status (low, middle, high, undefined) at the beginning of pregnancy

During the study period, the proportions of women belonging to different SES classes changed slightly. For instance, the proportion of middle-SES-class women increased during the years 2016–2018—from 43.1% in 2015 to 49.4% in 2018 (Fig. 3). At the same time, a decrease was seen in the number of women of undefined-SES class. The proportion of high-SES-class women increased from 18.6% in 2017 to 24.2% in 2018.

## Discussion

In our nationwide study, we found that women who smoked during pregnancy used labor analgesia more often than those who did not smoke during pregnancy. Furthermore, we found that SES did not have a clear impact on labor analgesia.

Räisänen et al. demonstrated that women who smoked during pregnancy used epidural analgesia more often than non-smoking women [8]. Our study showed that this trend was also seen in the use of other labor analgesia, particularly the difference between women who continued smoking after the first trimester and non-smoking women in the use of nitrous oxide, epidural, and non-medical analgesia.

Smoking has been shown to increase placental problems, such as impaired placental vasculature and metabolism, and to increase the risk for complications during childbirth [5, 20]. Due to these complications, the sensation of pain may increase. Also, smokers seem to have a higher need for pain relief after surgery [21] and the risk for chronic pain is higher in smokers than in non-smokers [22–24], which might indicate that smokers have a higher pain sensitivity.

A recent study found an association between smoking and central sensitization in women [25] and this might be one of the reasons why labor analgesia is used more by women who smoke. As women who smoke have a greater need for analgesia during labor, this condition should be recognized in clinical practice, and its underlying etiology should be further researched. This finding may also motivate women to quit smoking during pregnancy.

In our study, we did not find major differences between SES classes in the use of labor analgesia. A Swedish study stated that the use of epidural is impacted by local clinical practices, women’s wishes, and background [26] and another Swedish study demonstrated that epidural is more often used by women of lower SES class [11]. As the Swedish healthcare system is similar to the Finnish healthcare system, it is surprising that our study did not have findings in common with those from Sweden. Also, a study from Scotland with publicly funded healthcare found that lower SES positioning indicated lower use of epidural, even when the use of epidural was advisable [27]. Hence, further studies are needed to determine the reasons for these differences.

Other studies conclude that the ethnicity and educational level of the mother, as well as socioeconomic issues, affect the use of labor analgesia [28–30]. In Finland, all women have the same opportunity to access prenatal care and labor analgesia because of its publicly funded healthcare system and fairly uniform clinical practices. In addition, giving birth is practically free of charge for everyone in Finland, including immigrants and refugees. These aspects highlight the equality of Finnish health care.

One of the main strengths of this study is the use of a national MBR with high coverage and quality. Also, the data contained information from a period of 15 years, and therefore the dataset can be considered large. As for the main limitation, the data do not include all the attempted analgesia methods because only successful analgesia methods are reported to the register. Also, the smoking status was self-reported and not measured objectively, so there is a possibility of bias. Another limitation is that the register does not have information on the analgesic dose used, and we were not able to analyze possible differences between the groups. Furthermore, we had only information on intrapartum analgesia due to the register, and therefore we did not analyze postpartum analgesia. In addition, 26.0% of the pregnancies were excluded because of the missing SES class, but the number of women in the undefinable SES class was moderate, ranging from 8.6% to 21.0%.

## Conclusion

Our findings suggest that women who continued smoking after the first trimester used labor analgesia more often than non-smoking women. The etiology behind the increased use of pain relief among smoking women should be further researched. These results highlight the importance of reducing maternal smoking. We did not observe clinically important differences in the use of labor analgesia among women belonging to different SES classes, which indicates that Finnish universal healthcare promotes equality in labor analgesia.

## Data Availability

Data is not available due to Finnish research laws. Contact the Finnish Data Authority Findata for further information via e-mail: info@findata.fi.
